# The genome sequence of *Umbilicaria deusta* (L. Baumg., 1790) (Umbilicariales: Umbilicariaceae)

**DOI:** 10.12688/wellcomeopenres.27481.1

**Published:** 2026-09-03

**Authors:** Rebecca Yahr, Nathan A. M. Chrismas, Royal Botanic Garden Edinburgh Genome Acquisition Lab, Darwin Tree of Life Barcoding collective, Wellcome Sanger Institute Tree of Life Management, Samples and Laboratory team, Wellcome Sanger Institute Scientific Operations: Sequencing Operations, Wellcome Sanger Institute Tree of Life Core Informatics team, Tree of Life Core Informatics collective, Darwin Tree of Life Consortium

**Affiliations:** 1 Royal Botanic Garden Edinburgh, Edinburgh, Scotland, UK

**Keywords:** Umbilicaria deusta, Peppered Rock Tripe, genome sequence, chromosomal, Umbilicariales

## Abstract

We present a genome assembly of *Umbilicaria deusta* (Peppered Rock Tripe; Ascomycota; Lecanoromycetes; Umbilicariales; Umbilicariaceae). The genome sequence has a total length of 40.76 megabases. Most of the assembly (95.36%) is scaffolded into 24 chromosomal pseudomolecules. The mitochondrial genome has been assembled, with a length of 100.51 kilobases. This assembly was generated as part of the Darwin Tree of Life project, which produces reference genomes for eukaryotic species found in Britain and Ireland.

## Species taxonomy

Eukaryota; Opisthokonta; Fungi; Dikarya; Ascomycota; saccharomyceta; Pezizomycotina; leotiomyceta; Lecanoromycetes; OSLEUM clade; Umbilicariomycetidae; Umbilicariales; Umbilicariaceae; *Umbilicaria*; *Umbilicaria deusta* (L. Baumg., 1790) (NCBI:txid87276).

## Background

**Figure 1.**
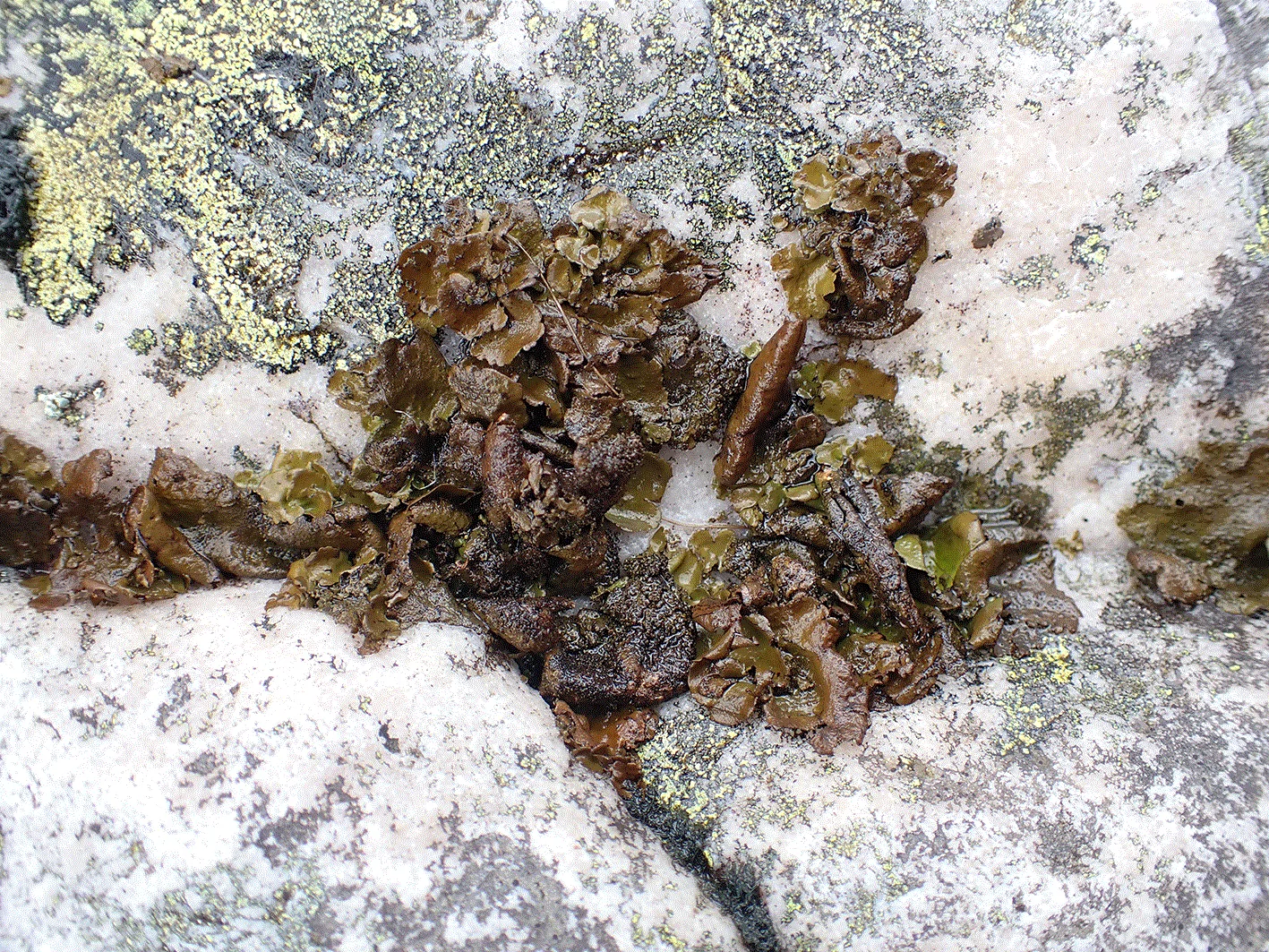
*Umbilicaria deusta* (R. Yahr 6739) in situ at Beinn Eighe NNR, after a rain shower.

*Umbilicaria deusta* ( Figure 1) is a lichenised member of the Umbilicariaceae, a family of lichens ranging from small microlichens to large leafy species in the class Lecanoromycetes ( Bendiksby & Timdal, 2013). The genus comprises rock-dwelling, high-altitude and high-latitude species ( Davydov *et al.*, 2024), recognised for their sometimes large and leafy thalli. Some members of the genus are used as food, although they are known to contain high concentrations of bitter lichen acids and are considered either delicacies or survival foods ( Richardson, 1991). *U. deusta* is an upland to montane, circumboreal species that tolerates slightly eutrophic conditions ( Martellos *et al.*, 2023) and can grow in dense swards atop low rocks, especially in moist areas ( Cannon *et al.*, 2024).

Lichens in the genus *Umbilicaria* form perennial leafy structures and associate with one or more co-occurring photobionts in the genus *Trebouxia* ( Paul *et al.*, 2018). At least one species can switch to yeast-like growth in culture, making it amenable to genetic transformation and development as a model system (e.g. Park *et al.*, 2013).

The genome of *Umbilicaria deusta* was sequenced as part of the Darwin Tree of Life Project, a collaborative effort to sequence all named eukaryotic species in the Atlantic Archipelago of Britain and Ireland. *U. deusta* has asexual, lichenised propagules and can form dense swards covering the tops of rocks. Here, we present a chromosomally complete genome sequence for *Umbilicaria deusta*, based on one collection from a rock beside a lochan at about 530 m elevation on Beinn Eighe, Kinlochewe, West Ross and Cromarty.

Other scaffold-level assemblies for this species are also available (GCA_053478105.1, GCA_023184635.1 and GCA_053478145.1), submitted by Senckenberg Gesellschaft für Naturforschung.

## Methods

### Sample acquisition and DNA barcoding

A specimen of *Umbilicaria deusta* (specimen ID EDTOL06319, ToLID glUmbDeus1) was used for genome sequencing. It was collected from Kinlochewe, Beinn Eighe Nature Reserve, West Ross and Cromarty, Scotland, UK (latitude 57.6157, longitude −5.3668) on 2023-07-18. The specimen was collected by Rebecca Yahr and Nathan Chrismas and identified by Rebecca Yahr (voucher R. Yahr 6739 deposited in herbarium E).

The initial identification was verified by DNA barcoding according to the framework developed by Twyford *et al*. (2024). DNA was extracted from the growing edges of fresh material with the use of a Red Extract’n’Amp extraction kit ( Jones *et al.*, 2024) . The internal transcribed spacer (ITS) region was amplified by PCR, and sequences compared to those sequences available on Genbank and UNITE.

### Nucleic acid extraction

Protocols for high molecular weight (HMW) DNA extraction developed at the Wellcome Sanger Institute (WSI) Tree of Life Core Laboratory are available on protocols.io ( Howard *et al.*, 2025). The glUmbDeus1 sample was weighed and triaged to determine the appropriate extraction protocol. For HMW DNA extraction, 85.0 mg of thallus tissue was homogenised by cryogenic disruption using the Covaris cryoPREP® Automated Dry Pulverizer. HMW DNA was extracted using the Manual Plant MagAttract v4 protocol. We used centrifuge-mediated fragmentation to produce DNA fragments in the 8–10 kb range, following the Covaris g-TUBE protocol for ultra-low input (ULI). Sheared DNA was purified by automated SPRI (solid-phase reversible immobilisation), using AMPure PB beads (Pacific Biosciences) and the Thermo Fisher KingFisher™ Apex to eliminate shorter fragments and concentrate the DNA. The concentration of the sheared and purified DNA was assessed using a Nanodrop spectrophotometer and Qubit Fluorometer using the Qubit dsDNA High Sensitivity Assay kit. Fragment size distribution was evaluated by running the sample on the FemtoPulse system. For this sample, the final post-shearing DNA had a Qubit concentration of 1.98 ng/μL and an average fragment size of 7.4 kb.

### PacBio HiFi library preparation and sequencing

Library preparation and sequencing were performed at the WSI Scientific Operations core. Prior to library preparation, the DNA was fragmented to ~10 kb. Ultra-low-input (ULI) libraries were prepared using the PacBio SMRTbell® Express Template Prep Kit 2.0 and gDNA Sample Amplification Kit. Samples were normalised to 20 ng DNA. Single-strand overhang removal, DNA damage repair, and end-repair/A-tailing were performed according to the manufacturer’s instructions, followed by adapter ligation. A 0.85× pre-PCR clean-up was carried out with Promega ProNex beads.

The DNA was evenly divided into two aliquots for dual PCR (reactions A and B), both following the manufacturer’s protocol. A 0.85× post-PCR clean-up was performed with ProNex beads. DNA concentration was measured using a Qubit Fluorometer v4.0 (Thermo Fisher Scientific) with the Qubit HS Assay Kit, and fragment size was assessed on an Agilent Femto Pulse Automated Pulsed Field CE Instrument (Agilent Technologies) using the gDNA 55 kb BAC analysis kit. PCR reactions A and B were then pooled, ensuring a total mass of ≥500 ng in 47.4 μl.

The pooled sample underwent another round of DNA damage repair, end-repair/A-tailing, and hairpin adapter ligation. A 1× clean-up was performed with ProNex beads, followed by DNA quantification using the Qubit and fragment size analysis using the Agilent Femto Pulse. Size selection was performed on the Sage Sciences PippinHT system, with target fragment size determined by Femto Pulse analysis (typically 4–9 kb). Size-selected libraries were cleaned with 1.0× ProNex beads and normalised to 2 nM before sequencing.

The sample was sequenced on a Revio instrument (Pacific Biosciences, California, USA). Prepared libraries with a molarity greater than 2 nM were normalised to 2 nM, and 15 μL was used for making complexes. For libraries with a molarity below 2 nM, the available 10 μL library volume was used without normalisation. Primers were annealed and polymerases were bound to generate circularised complexes, following the manufacturer’s instructions. Complexes were purified using 1.2× SMRTbell beads, diluted to the Revio loading concentration of 200–300 pM, and spiked with a Revio sequencing internal control. The sample was sequenced on a Revio 25M SMRT cell. SMRT Link software (Pacific Biosciences), a web-based workflow manager, was used to configure and monitor the run and to carry out primary and secondary data analysis.

### Hi-C

***Sample preparation and crosslinking***

Hi-C data were generated from the thallus tissue of glUmbDeus1 using the Arima-HiC v2 kit (Arima Genomics). Tissue was finely ground using the Covaris cryoPREP Dry Pulverizer (Covaris), and then subjected to nuclei isolation. Nuclei were isolated using a modified protocol based on the Qiagen QProteome Cell Compartment Kit (Qiagen), in which only the Lysis and CE2 buffers were used, with QIAshredder spin columns. After isolation, nuclei were fixed using formaldehyde to a final concentration of 2% to crosslink the DNA. The crosslinked DNA was then digested and biotinylated according to the manufacturer’s instructions. A clean-up step was performed with SPRIselect beads before library preparation. DNA concentration was quantified using the Qubit Fluorometer v4.0 (Thermo Fisher Scientific) and the Qubit HS Assay Kit, following the manufacturer’s instructions.

***Hi-C library preparation and sequencing***

Biotinylated DNA constructs were fragmented using a Covaris E220 sonicator and size selected to 400–600 bp using SPRISelect beads. DNA was enriched with Arima-HiC v2 kit Enrichment beads. End repair, A-tailing, and adapter ligation were carried out with the NEBNext Ultra II DNA Library Prep Kit (New England Biolabs), following a modified protocol where library preparation occurs while DNA remains bound to the Enrichment beads. Library amplification was performed using KAPA HiFi HotStart mix and a custom Unique Dual Index (UDI) barcode set (Integrated DNA Technologies). Depending on sample concentration and biotinylation percentage determined at the crosslinking stage, libraries were amplified with 10–16 PCR cycles. Post-PCR clean-up was performed with SPRISelect beads. Libraries were quantified using the AccuClear Ultra High Sensitivity dsDNA Standards Assay Kit (Biotium) and a FLUOstar Omega plate reader (BMG Labtech).

Prior to sequencing, libraries were normalised to 10 ng/μL. Normalised libraries were quantified again to create equimolar and/or weighted 2.8 nM pools. Pool concentrations were checked using the Agilent 4200 TapeStation (Agilent) with High Sensitivity D500 reagents before sequencing. Sequencing was performed using paired-end 150 bp reads on the Illumina NovaSeq X.

### Genome assembly

Prior to assembly of the PacBio HiFi reads, a database of *k*-mer counts ( *k* = 31) was generated from the filtered reads using FastK. GenomeScope2 ( Ranallo-Benavidez *et al.*, 2020) was used to analyse the *k*-mer frequency distributions. 

The HiFi reads were assembled using Hifiasm ( Cheng *et al.*, 2021) with the --primary option. Haplotypic duplications were identified and removed using purge_dups ( Guan *et al.*, 2020). The Hi-C reads ( Rao *et al.*, 2014) were mapped to the primary contigs using bwa-mem2 ( Vasimuddin *et al.*, 2019), and the contigs were scaffolded in YaHS ( Zhou *et al.*, 2023) with the --break option for handling potential misassemblies. The scaffolded assemblies were evaluated using Gfastats ( Formenti *et al.*, 2022), BUSCO ( Manni *et al.*, 2021) and MerquryFK ( Rhie *et al.*, 2020).

The mitochondrial genome was assembled using MitoHiFi ( Uliano-Silva *et al.*, 2023) and OATK ( Zhou *et al.*, 2025). The comparator for the mitochondrial assembly was the mitochondrial genome of *Ramalina intermedia* (NC_044483.1).

The same sequencing datasets were also used to generate the unbinned lichen metagenome assembly glUmbDeus1.metagenome.1, together with associated metagenome-assembled genomes and bins, available under ENA BioProject PRJEB88595.

### Assembly curation

The assembly was decontaminated using the Assembly Screen for Cobionts and Contaminants ( ASCC) pipeline. TreeVal was used to generate the flat files and maps for use in curation. Manual curation was conducted primarily in PretextView and HiGlass ( Kerpedjiev *et al.*, 2018). Scaffolds were visually inspected and corrected as described by Howe *et al*. (2021). Manual corrections included ten breaks, seven joins, and removal of four haplotypic duplications. This reduced the scaffold count by 19.0% and reduced the scaffold N50 by 2.4%. The curation process is described at sanger-tol/curation-resources . PretextSnapshot was used to generate a Hi-C contact map of the final assembly.

### Assembly quality assessment

The MerquryFK tool ( Rhie *et al.*, 2020) was run in a Singularity container ( Kurtzer *et al.*, 2017) to evaluate assembly quality for the primary and alternate haplotypes using the *k*-mer database ( *k* = 31) computed prior to genome assembly. Because this database was derived from the mixed lichen-thallus read set, the resulting *k*-mer completeness estimates were not used to assess the fungal assembly.

The genome was analysed using the BlobToolKit pipeline, a Nextflow implementation of the earlier Snakemake version ( Challis *et al.*, 2020). PacBio reads were aligned to the assembly using minimap2 ( Li, 2018) and processed with SAMtools ( Danecek *et al.*, 2021) to generate coverage tracks. The pipeline runs BUSCO ( Manni *et al.*, 2021) using lineages identified from the NCBI Taxonomy ( Schoch *et al.*, 2020). For the three basal BUSCO lineages, eukaryota, bacteria and archaea, BUSCO genes are aligned to the UniProt Reference Proteomes database ( Bateman *et al.*, 2023) using DIAMOND BLASTp ( Buchfink *et al.*, 2021). The genome assembly is divided into chunks based on the density of BUSCO genes from the closest taxonomic lineage, and each chunk is aligned to the UniProt Reference Proteomes database using DIAMOND BLASTx. Sequences without DIAMOND BLASTx hits are extracted using seqtk and aligned to the NT database using BLASTn ( Altschul *et al.*, 1990). The outputs are consolidated into a BlobDir for visualisation in BlobToolKit. The BlobToolKit pipeline was developed using nf-core tooling ( Ewels *et al.*, 2020) and MultiQC ( Ewels *et al.*, 2016), with containerisation through Docker ( Merkel, 2014) and Singularity ( Kurtzer *et al.*, 2017).

## Genome sequence report

### Sequence data

The genome of a specimen of *Umbilicaria deusta* was sequenced using Pacific Biosciences single-molecule HiFi long reads, generating 66.69 Gb (gigabases) from 8.18 million reads, which were used to assemble the genome. Hi-C sequencing produced 82.01 Gb from 271.55 million read pairs, which were used to scaffold the assembly. Table 1 summarises the specimen and sequencing details.

**Table 1.** Specimen and sequencing data for *Umbilicaria deusta* (BioProject PRJEB81838).

Platform	PacBio HiFi	Hi-C
**ToLID**	glUmbDeus1	glUmbDeus1
**Specimen ID**	EDTOL06319	EDTOL06319
**BioSample (source individual)**	SAMEA114749973	SAMEA114749973
**BioSample (tissue)**	SAMEA114763994	SAMEA114788253
**Tissue**	thallus	thallus
**Instrument**	Revio	Illumina NovaSeq X
**Run accessions**	ERR13917198	ERR13925930
**Read count total**	8.18 million reads	271.55 million read pairs
**Base count total**	66.69 Gb	82.01 Gb

### Assembly statistics

The primary haplotype was assembled, and contigs corresponding to an alternate haplotype were also deposited in INSDC databases. The final assembly has a total length of 40.76 Mb in 97 scaffolds, with 28 gaps, and a scaffold N50 of 1.69 Mb ( Table 2).

**Table 2.** Genome assembly data for *Umbilicaria deusta.*

Genome assembly	Primary assembly
**Assembly name**	glUmbDeus1.1
**Assembly accession**	GCA_964340765.1
**Alternate haplotype accession**	GCA_964340595.1
**Assembly level**	chromosome
**Span (Mb)**	40.76
**Number of chromosomes**	24
**Number of contigs**	125
**Contig N50 (Mb)**	1.35
**Number of scaffolds**	97
**Scaffold N50 (Mb)**	1.69
**Organelles**	Mitochondrial genome: 100.51 kb

**Figure 2.**
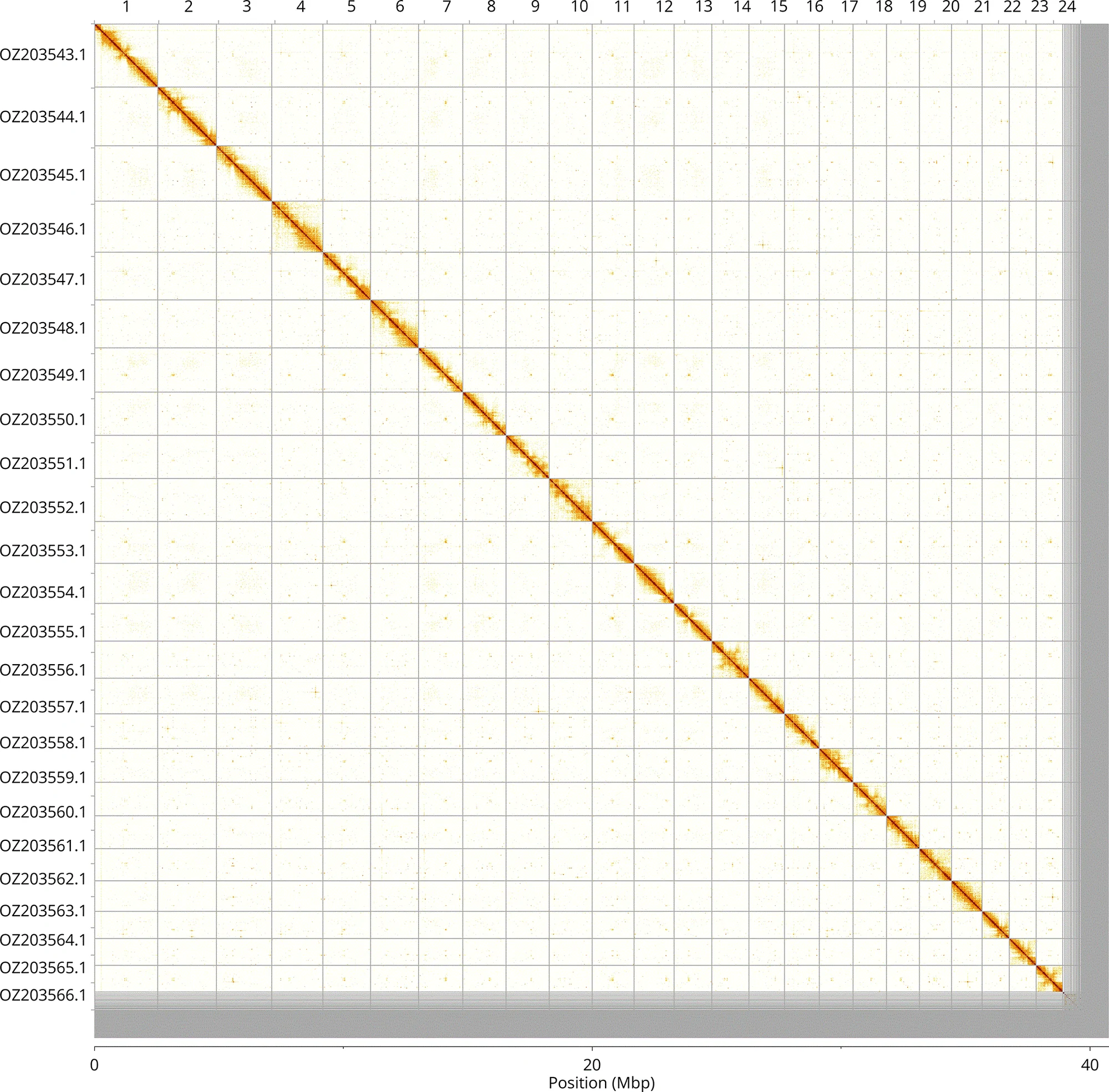
Hi-C contact map of the *Umbilicaria deusta* genome assembly. Assembled chromosomes are shown in order of size and labelled along the axes, with a megabase scale shown below. The plot was generated using PretextSnapshot.

Most of the assembly sequence (95.36%) was assigned to 24 chromosomal-level scaffolds. These chromosome-level scaffolds, confirmed by Hi-C data, are named according to size ( Figure 2; Table 3). The mitochondrial genome was also assembled (length 100.51 kb, OZ203567.1). This sequence is included as a contig in the multifasta file of the genome submission and as a standalone record.

**Table 3.** Chromosomal pseudomolecules in the primary genome assembly of *Umbilicaria deusta* glUmbDeus1.

INSDC accession	Molecule	Length (Mb)	GC%
OZ203543.1	1	2.54	48
OZ203544.1	2	2.35	47
OZ203545.1	3	2.22	49
OZ203546.1	4	2.05	44
OZ203547.1	5	1.92	46.50
OZ203548.1	6	1.92	46
OZ203549.1	7	1.78	48
OZ203550.1	8	1.73	48.50
OZ203551.1	9	1.73	47.50
OZ203552.1	10	1.73	47
OZ203553.1	11	1.69	48
OZ203554.1	12	1.60	48.50
OZ203555.1	13	1.52	49.50
OZ203556.1	14	1.49	47
OZ203557.1	15	1.44	49
OZ203558.1	16	1.39	47.50
OZ203559.1	17	1.36	46
OZ203560.1	18	1.35	46.50
OZ203561.1	19	1.31	47
OZ203562.1	20	1.30	48
OZ203563.1	21	1.22	48
OZ203564.1	22	1.09	48
OZ203565.1	23	1.08	48
OZ203566.1	24	1.07	47.50

### Assembly quality metrics

**Figure 3.**
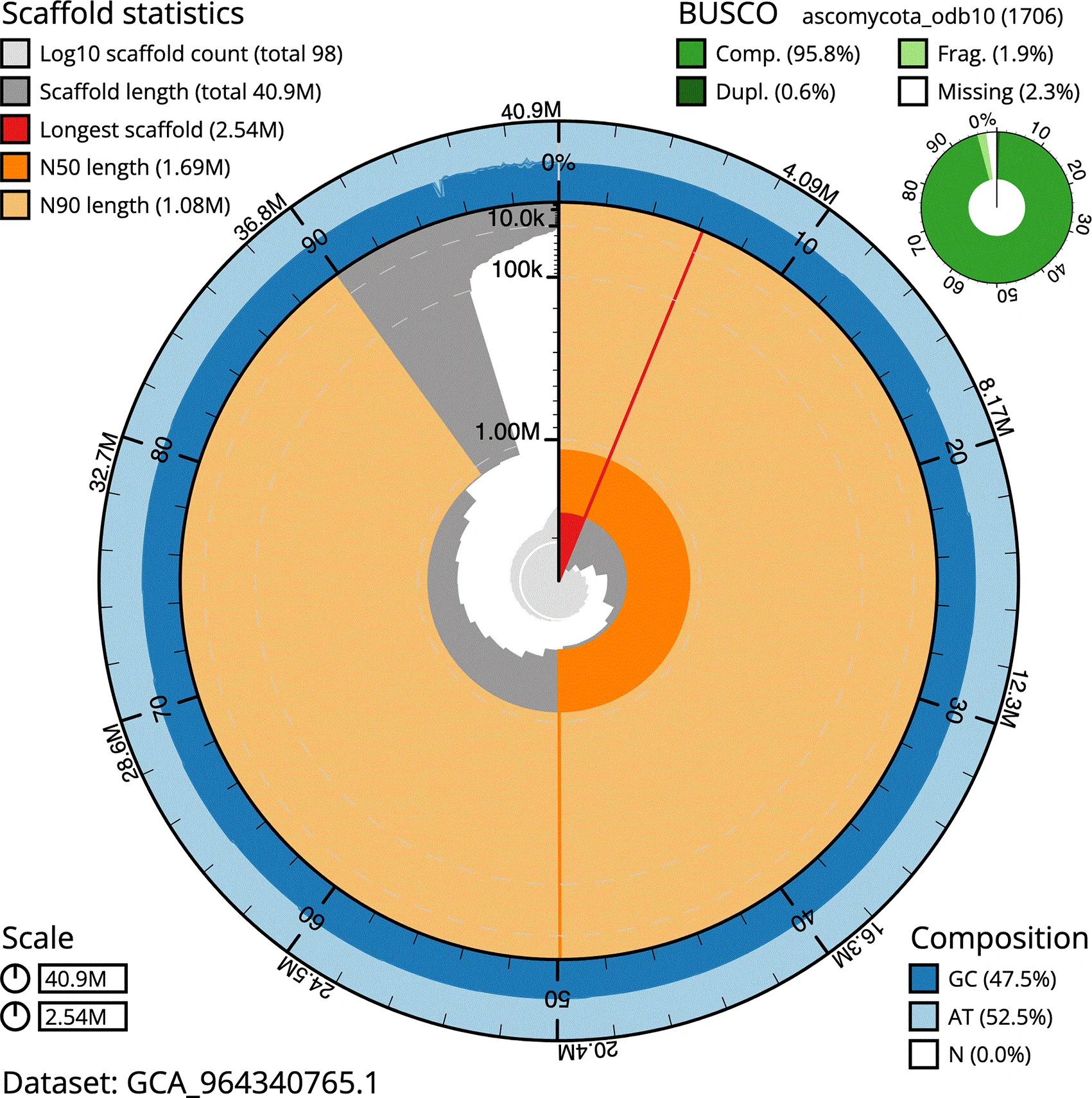
Assembly metrics for glUmbDeus1.1. The BlobToolKit snail plot provides an overview of assembly metrics and BUSCO gene completeness. The circumference represents the length of the whole genome sequence, and the main plot is divided into 1 000 bins around the circumference. The outermost blue tracks display the distribution of GC, AT, and N percentages across the bins. Scaffolds are arranged clockwise from longest to shortest and are depicted in dark grey. The longest scaffold is indicated by the red arc, and the deeper orange and pale orange arcs represent the N50 and N90 lengths. A light grey spiral at the centre shows the cumulative scaffold count on a logarithmic scale. A summary of complete, fragmented, duplicated, and missing BUSCO genes in the ascomycota_odb10 set is presented at the top right. An interactive version of this figure can be accessed on the BlobToolKit viewer.

**Figure 4.**
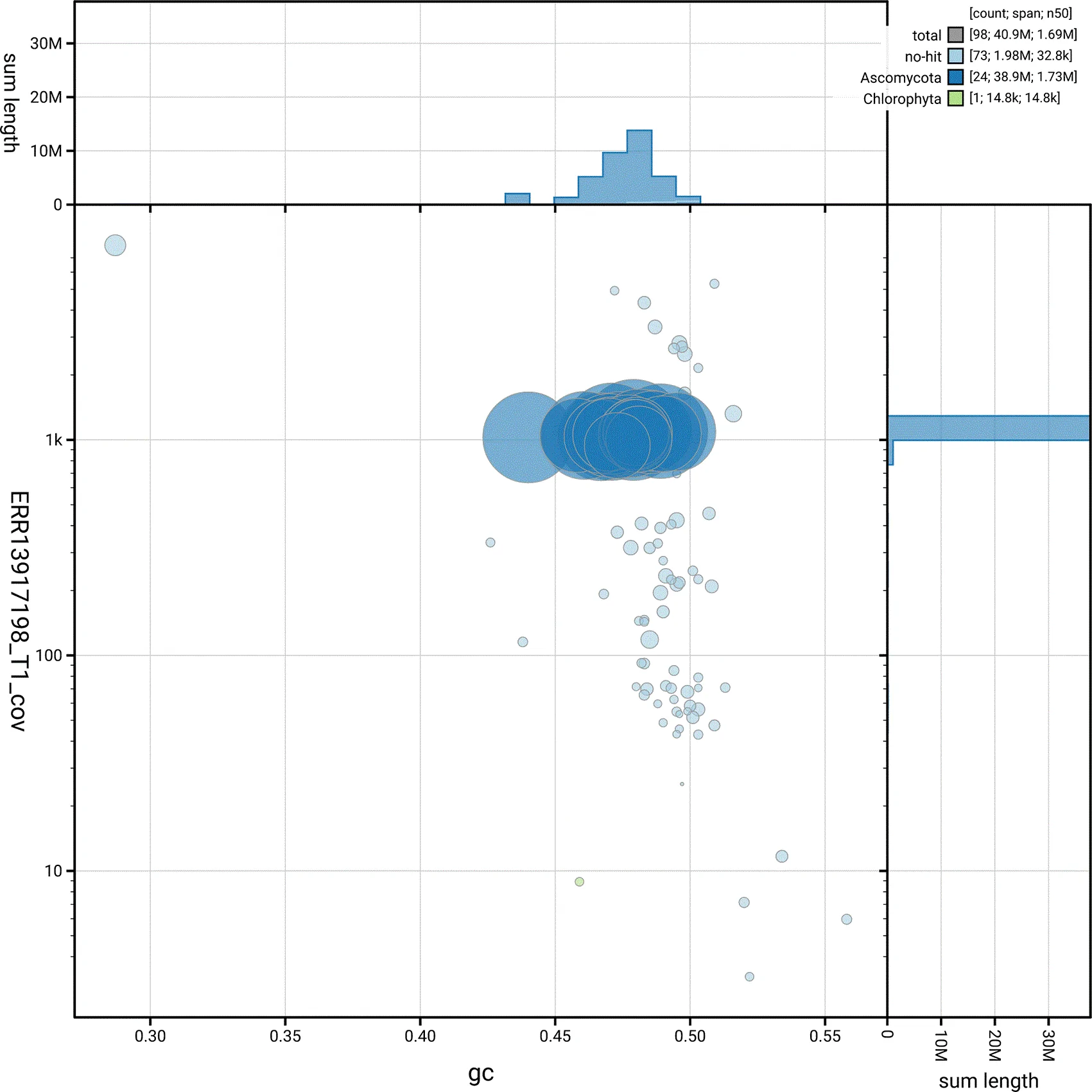
BlobToolKit blob plot for glUmbDeus1.1. The plot shows base coverage (vertical axis) and GC content (horizontal axis). The circles represent scaffolds, with the size proportional to scaffold length and the colour representing phylum membership. The histograms along the axes display the total length of sequences distributed across different levels of coverage and GC content. An interactive version of this figure is available on the BlobToolKit viewer.

The combined primary and alternate assemblies achieve an estimated QV of 56.3 in MerquryFK. BUSCO v.5.5.0 analysis using the ascomycota_odb10 reference set ( *n* = 1,706) identified 95.8% of the expected gene set (single = 95.2%, duplicated = 0.6%). The snail plot in Figure 3 summarises the scaffold length distribution and other assembly statistics for the primary assembly. The blob plot in Figure 4 shows the distribution of scaffolds by GC proportion and coverage.

Table 4 lists the assembly metric benchmarks adapted from Rhie *et al.* (2021) and https://www.earthbiogenome.org/report-on-assembly-standards . The EBP metric calculated for primary assembly is **6.C.Q59**, meeting the recommended 6.C.Q40 reference standard.

**Table 4.** Earth Biogenome Project summary metrics for the *Umbilicaria deusta* assembly.

Measure	Value	Benchmark
EBP summary (primary)	6.C.Q59	6.C.Q40
Contig N50 length	1.35 Mb	≥ 1 Mb
Scaffold N50 length	1.69 Mb	= chromosome N50
Consensus quality (QV)	Primary: 59.7; alternate: 48.5; combined: 56.3	≥ 40
BUSCO	C:95.8% [S:95.2%, D:0.6%], F:1.9%, M:2.3%, n:1,706	S > 90%; D < 5%
Percentage of assembly assigned to chromosomes	95.36%	≥ 90%

*Notes:* The EBP summary uses log10(Contig N50); chromosome-level (C) or log10(Scaffold N50); Q (Merqury QV). BUSCO: C=complete; S=single-copy; D=duplicated; F=fragmented; M=missing; n=orthologues.

**Table 5.** Software versions and sources used for *Umbilicaria deusta.*

Software	Version	Source
BLAST	2.14.0	https://ftp.ncbi.nlm.nih.gov/blast/executables/blast+/
BlobToolKit	4.3.9	https://github.com/genomehubs/blobtoolkit
blobtk	0.5.1	https://github.com/genomehubs/blobtk
BUSCO	5.5.0; 6.1.0	https://gitlab.com/ezlab/busco
bwa-mem2	2.2.1	https://github.com/bwa-mem2/bwa-mem2
DIAMOND	2.1.8	https://github.com/bbuchfink/diamond
fasta_windows	0.2.4	https://github.com/tolkit/fasta_windows
FastK	1.1	https://github.com/thegenemyers/FASTK
GenomeScope2.0	2.0.1	https://github.com/tbenavi1/genomescope2.0
Gfastats	1.3.6	https://github.com/vgl-hub/gfastats
Hifiasm	0.19.8-r603	https://github.com/chhylp123/hifiasm
HiGlass	1.13.4	https://github.com/higlass/higlass
MerquryFK	1.1.0-c1	https://github.com/thegenemyers/MERQURY.FK
Minimap2	2.24-r1122; 2.29-r1283	https://github.com/lh3/minimap2
MitoHiFi	3.2.2	https://github.com/marcelauliano/MitoHiFi
Oatk	1.0	https://github.com/c-zhou/oatk
MultiQC	1.14; 1.17 and 1.18	https://github.com/MultiQC/MultiQC
Nextflow	23.10.0; 25.10.0; 25.10.4	https://github.com/nextflow-io/nextflow
PretextSnapshot	0.0.6	https://github.com/sanger-tol/PretextSnapshot
PretextView	1.0.3	https://github.com/sanger-tol/PretextView
purge_dups	1.2.3	https://github.com/dfguan/purge_dups
samtools	1.17; 1.18; 1.19.2; 1.23.1; 1.6	https://github.com/samtools/samtools
sanger-tol/ascc	0.1.0	https://github.com/sanger-tol/ascc
sanger-tol/blobtoolkit	0.6.0	https://github.com/sanger-tol/blobtoolkit
sanger-tol/curationpretext	1.4.2	https://github.com/sanger-tol/curationpretext
Seqtk	1.4-r122	https://github.com/lh3/seqtk
Singularity	3.9.0	https://github.com/sylabs/singularity
TreeVal	1.1.1	https://github.com/sanger-tol/treeval
YaHS	1.2a.2	https://github.com/c-zhou/yahs

## Author information

• Members of the Royal Botanic Garden Edinburgh Genome Acquisition Lab
• Members of the Darwin Tree of Life Barcoding collective
• Members of the Wellcome Sanger Institute Tree of Life Management, Samples and Laboratory team
• Members of Wellcome Sanger Institute Scientific Operations – Sequencing Operations
• Members of the Wellcome Sanger Institute Tree of Life Core Informatics team
• Members of the Tree of Life Core Informatics collective
• Members of the Darwin Tree of Life Consortium

## Wellcome Sanger Institute – Legal and Governance

The materials that have contributed to this genome note have been supplied by a Darwin Tree of Life Partner. The submission of materials by a Darwin Tree of Life Partner is subject to the **‘Darwin Tree of Life Project Sampling Code of Practice’**, which can be found in full on the Darwin Tree of Life website. By agreeing with and signing up to the Sampling Code of Practice, the Darwin Tree of Life Partner agrees they will meet the legal and ethical requirements and standards set out within this document in respect of all samples acquired for, and supplied to, the Darwin Tree of Life Project. Further, the Wellcome Sanger Institute employs a process whereby due diligence is carried out proportionate to the nature of the materials themselves, and the circumstances under which they have been/are to be collected and provided for use. The purpose of this is to address and mitigate any potential legal and/or ethical implications of receipt and use of the materials as part of the research project, and to ensure that in doing so we align with best practice wherever possible. The overarching areas of consideration are:

• Ethical review of provenance and sourcing of the material
• Legality of collection, transfer and use (national and international)

Each transfer of samples is further undertaken according to a Research Collaboration Agreement or Material Transfer Agreement entered into by the Darwin Tree of Life Partner, Genome Research Limited (operating as the Wellcome Sanger Institute), and in some circumstances, other Darwin Tree of Life collaborators.

## Data Availability

European Nucleotide Archive: Umbilicaria deusta. Accession number PRJEB81838; https://identifiers.org/ena.embl/PRJEB81838. The genome sequence is released openly for reuse. The *Umbilicaria deusta* genome sequencing initiative is part of the Darwin Tree of Life Project ( PRJEB40665), Aquatic Symbiosis Genomics Project ( PRJEB43743) and Sanger Institute Tree of Life Programme ( PRJEB43745). The same sequencing datasets were also used to generate the unbinned lichen metagenome assembly glUmbDeus1.metagenome.1, together with associated metagenome-assembled genomes and bins, available under ENA BioProject PRJEB88595. All raw sequence data and the assembly have been deposited in INSDC databases. The genome will be annotated using available RNA-Seq data and presented through the Ensembl pipeline at the European Bioinformatics Institute. Raw data and assembly accession identifiers are reported in Tables 1 and 2. Pipelines used for genome assembly at the WSI Tree of Life are available at https://pipelines.tol.sanger.ac.uk/pipelines. Table 5 lists software versions used in this study.
