# Supplementary material for: The genome sequence of Umbilicaria deusta (L. Baumg., 1790) (Umbilicariales: Umbilicariaceae)
Source: Wellcome Open Res. 2026 Sep 3;11:573. [Version 1] doi: 10.12688/wellcomeopenres.27481.1 (PMC13615350; doi:10.12688/wellcomeopenres.27481.1)
Supplement: Peer review history

# Reviewer report: Reviewer response for version 1

**DOI:** 10.21956/wellcomeopenres.30248.r167192

**Published:** 2026-09-25

**Authors:** Sarah E. Calvo

**Affiliations:** 1 Broad Institute of Massachusetts Institute of Technology and Harvard, Cambridge, UK

Yahr and colleagues collect and present a genome assembly for *Umbilicaria deusta* (Peppered Rock Tripe), generated under the Darwin Tree of Life project using standard PacBio HiFi long-read sequencing combined with Hi-C scaffolding. The assembly totals 40.76 Mb across 97 scaffolds, with 95.36% of sequence assigned to 24 chromosomal pseudomolecules and a 100.51 kb mitochondrial genome assembled separately. The reported metrics show high quality consistent with other Darwin Tree of Life projects, based on BUSCO, Merqury, and an EBP designation of 6.C.Q59 — exceeding the recommended standard. The bioinformatics pipeline (Hifiasm, purge_dups, YaHS, MitoHiFi/OATK, BlobToolKit, manual PretextView/HiGlass curation) follows well-established, widely used community standards for this consortium, and the methods are described in sufficient detail — including specific kit names, bead ratios, cycle numbers, and software versions — to permit replication. The data appear to be high quality and were publicly available and easy to access. The work of the authors toward this important scientific goal is appreciated.

**Critiques:**

- The limitation of using a single specimen was explicitly acknowledged, as was the use of thallus tissue instead of axenic culture — but the language was very technical and might benefit from a higher-level sentence or two explaining to readers the difficulty, for lichen assembly, of distinguishing reads that map to the fungus versus the photobiont.
- Regarding sequencing depth relative to genome size: 66.69 Gb of HiFi data against a final ~41 Mb fungal genome implies extremely high raw coverage nominally, but a substantial fraction of that data is non-target (photobiont), and the mixed-sample nature makes true fungal-specific coverage depth non-obvious from the text. Could this be stated explicitly, similar to the point above? I think it could benefit from stating that the assembly of the fungal species *U. deusta* is ~41 Mb (X mean coverage), whereas reads mapping to other species (photobiont/cobiont) accounted for X Mb (Y mean coverage).
- I was confused by the statement "Other scaffold-level assemblies for this species are also available (GCA_053478105.1, GCA_023184635.1 and GCA_053478145.1), submitted by Senckenberg Gesellschaft für Naturforschung." It seemed to imply this is a chromosome-level assembly (although there are many more contigs than chromosomes) and did not indicate how the new assembly outperforms the pre-existing scaffold assemblies. This is not required to address but was a point of confusion to me.

Overall, this work adds an important resource to the field. Thank you for your work.

Are sufficient details of methods and materials provided to allow replication by others?

Yes

Is the rationale for creating the dataset(s) clearly described?

Yes

Are the datasets clearly presented in a useable and accessible format?

Yes

Are the protocols appropriate and is the work technically sound?

Yes

Reviewer Expertise:

Fungal genome annotation, Acanthamoeba genome annotation, mitochondrial biology

I confirm that I have read this submission and believe that I have an appropriate level of expertise to confirm that it is of an acceptable scientific standard.

# Reviewer report: Reviewer response for version 1

**DOI:** 10.21956/wellcomeopenres.30248.r167198

**Published:** 2026-09-23

**Authors:** Jason Stajich

**Affiliations:** 1 University of California Riverside, Riverside, California, USA

This standardized genome report is complete in its description of data acquisition, processing and QC, assembly, and evaluation.  No concerns about the report or the description of the sample collecting, permits, or accessioning of original material.

 The figures add nice context for the organism itself, data quality, completeness and summary statistics.

 Data are public, raw and assembly accessible.

Are sufficient details of methods and materials provided to allow replication by others?

Yes

Is the rationale for creating the dataset(s) clearly described?

Yes

Are the datasets clearly presented in a useable and accessible format?

Yes

Are the protocols appropriate and is the work technically sound?

Yes

Reviewer Expertise:

mycology, genomics, evolutionary biology

I confirm that I have read this submission and believe that I have an appropriate level of expertise to confirm that it is of an acceptable scientific standard.
